# Linkage Disequilibrium Estimation of Effective Population Size with Immigrants from Divergent Populations: A Case Study on Spanish Mackerel (*Scomberomorus commerson*)

**DOI:** 10.1534/g3.112.005124

**Published:** 2013-04-01

**Authors:** Gilbert Michael Macbeth, Damien Broderick, Rik C. Buckworth, Jennifer R. Ovenden

**Affiliations:** * Molecular Fisheries Laboratory, Queensland Government, St Lucia, Queensland, 4072, Australia; † CSIRO Marine & Atmospheric Research, Wealth from Oceans National Research Flagship, Brisbane, Queensland, 4001, Australia

**Keywords:** effective population size, bias, nontarget populations, correspondence analysis, outliers

## Abstract

Estimates of genetic effective population size (*Ne*) using molecular markers are a potentially useful tool for the management of endangered through to commercial species. However, pitfalls are predicted when the effective size is large because estimates require large numbers of samples from wild populations for statistical validity. Our simulations showed that linkage disequilibrium estimates of *Ne* up to 10,000 with finite confidence limits can be achieved with sample sizes of approximately 5000. This number was deduced from empirical allele frequencies of seven polymorphic microsatellite loci in a commercially harvested fisheries species, the narrow-barred Spanish mackerel (*Scomberomorus commerson*). As expected, the smallest SD of *Ne* estimates occurred when low-frequency alleles were excluded. Additional simulations indicated that the linkage disequilibrium method was sensitive to small numbers of genotypes from cryptic species or conspecific immigrants. A correspondence analysis algorithm was developed to detect and remove outlier genotypes that could possibly be inadvertently sampled from cryptic species or nonbreeding immigrants from genetically separate populations. Simulations demonstrated the value of this approach in Spanish mackerel data. When putative immigrants were removed from the empirical data, 95% of the *Ne* estimates from jacknife resampling were greater than 24,000.

The effective number in a breeding stock was defined by Wright (1930) as an idealized number of parents in a population that cause a given level of inbreeding or given change in allele frequencies. This effective number “is much smaller as a rule than the actual number of adult individuals” (Wright 1930) but is an important parameter in ecological studies because any change over time indicates underlying changes in population structure. The mean squared correlation of alleles at different loci is a measure of linkage disequilibrium, which can be used to estimate genetic effective population size $\left(\hat{N}e\right)$ of diploid individuals. In small populations there is a greater correlation of alleles between loci compared with larger populations (Pudovkin *et al.* 1996; Hedgecock *et al.* 2007; Zhdanova and Pudovkin 2008) and hence there is a relationship with genetic effective population size (Waples 2006). It was suggested by Waples and Do (2010) that strong assortative mating would lead to biases in $\hat{N}e$. Later, Waples and England (2011) investigated migration between populations and concluded that the linkage disequilibrium method was robust to equilibrium migration with $\hat{N}e$, reflecting that of the local subpopulation. Waples and England (2011) also showed that pulse migration of strongly divergent individuals was found to depress estimates of local *Ne*.

The effect of pulse migration is an important finding because related factors could also lead to depressed *Ne* estimates. These factors could include inadvertent sampling of nontarget species and sampling of the same species but from populations that have become genetically divergent over many generations. Some fish species are known to exhibit natal philopatry, in which individuals have home spawning grounds but later disperse. Examples include herring, cod, sharks, swordfish, and anadromous salmonids (Beacham *et al.* 2005; Bekkevold *et al.* 2007; Svedang *et al.* 2007; Jorgensen *et al.* 2009; Smith and Alvarado-Bremer 2010). Under this model, samples from a single location taken when the species was in the dispersed phase could represent several genetically distinct (*i.e.*, mixed) stocks. The samples would not represent a panmictic population, causing deviations from the expected linkage disequilibria and a bias in the linkage disequilibrium estimation of *Ne*. For example, a downward bias in *Ne* estimates was simulated by Palstra and Ruzzante (2011) when divergent populations were pooled.

The frequency of natal philopatry is poorly known across marine species and virtually unknown in Australian fisheries species (Blower *et al.* 2012; Tillett *et al.* 2012). A species of considerable fisheries interest in Australia, and much of the Indo-West Pacific, is the narrow-barred Spanish mackerel, *Scomberomorus commerson*. It is a large, fast-swimming pelagic predator found throughout tropical and subtropical neritic waters of the Indo-West Pacific (Collette and Nauen 1983). If *S. commerson* exhibit natal philopatry, the mixing of genetically distinct populations within the sample collection area could depress $\hat{N}e$ in a similar manner suggested by pulse migration (Waples and England 2011). Seasonal aggregation for spawning followed by dispersal is supported by several lines of evidence: (1) seasonal variations in the availability of *S. commerson* (Buckworth *et al.* 2007), (2) a tag release study in northern Australia showing dispersal of recaptured fish with 12% more than 600 nautical miles away (Buckworth *et al.* 2007), (3) movement of fish on the eastern Australian coast southwards in summer presumably for feeding (McPherson 1988), and (4) multiple genetically distinct stocks in Southeast Asia (Fauvelot and Borsa 2011). The species is under active management throughout its range in Australia, and accurate estimates of effective population size have the potential to assist (Hare *et al.* 2011; Luikart *et al.* 2010; Ovenden *et al.* 2007; Palstra and Ruzzante 2008).

In this work we document a case study of the pitfalls associated with the estimation of *Ne* in *S. commerson* when large samples of genotypes (*S* > 5000) were sampled from a single location in northern Australia. We compare the estimated *Ne* from simulated populations with those from the empirical data. We critically review the estimates of *Ne* by testing hypotheses that the sampled population is a mixed stock. We also develop a method of screening and removing individuals likely to be from nontarget populations or species.

## Materials and Methods

### Linkage disequilibrium estimation of effective population size ($\hat{N}e$)

Linkage disequilibrium estimation of effective population size is derived from the correlation of alleles between loci. The correlation is determined from allele frequencies and has the general form of the phi correlation coefficient

$${\hat{r}}_{A_{j}B_{k}}=\frac{{\hat{\Delta }}_{A_{j}B_{k}}}{\sqrt{\left[{\hat{p}}_{A_{j}}\left(1-{\hat{p}}_{A_{j}}\right)+{\hat{D}}_{A_{j}}\right]\left[{\hat{p}}_{B_{k}}\left(1-{\hat{p}}_{B_{k}}\right)+{\hat{D}}_{B_{k}}\right]}}$$

(Weir 1996, p137) where ${\hat{r}}_{A_{j}B_{k}}$ is the estimated correlation between the *j*^th^ allele in locus *A* and *k*^th^ allele in locus *B* given ${\hat{p}}_{A_{j}}$ is the empirical frequency estimation of allele *j* in locus *A*, ${\hat{p}}_{B_{k}}$ is the empirical frequency estimation of allele *k* in locus *B*, ${\hat{D}}_{A_{j}}=f\left(A_{j}A_{j}\right)-{{\hat{p}}_{A_{j}}}^{2}$ and ${\hat{D}}_{B_{k}}=f\left(B_{k}B_{k}\right)-{{\hat{p}}_{B_{k}}}^{2}$ represent the additional variance in allele frequencies due to deviations in Hardy Weinberg equilibrium, where *f*() in the aforementioned formulae denote the observed homozygote frequencies. When diploid genotypes are sampled, the gametic phase is unknown with linkage disequilibrium determined by the Burrows estimate ${\hat{\Delta }}_{A_{j}B_{k}}=\hat{p}\left(A_{j}B_{k}\right)-2{\hat{p}}_{A_{j}}{\hat{p}}_{B_{k}}$ (Schaid 2004). In this equation, ${\hat{\Delta }}_{A_{j}B_{k}}$ is the deviation from the estimated probability of *A_j_B_k_* gametes, $\hat{p}\left(A_{j}B_{k}\right)$, from their expected probability $2{\hat{p}}_{A_{j}}{\hat{p}}_{B_{k}}$. The value $\hat{p}\left(A_{j}B_{k}\right)$ had to be determined indirectly from the count of *A_j_B_k_* combinations within biallelic genotypes (Table 1) because the gamete frequencies *A_j_B_k_* were unknown. In Table 1, # indicates that there were no *A_j_B_k_* gametes present within the genotype; thus, the expected number of *A_j_B_k_* gametes given the genotype $A_{j}A_{j*}B_{k}B_{k*}$ is equal to $X_{A_{j},A_{j*},B_{k},B_{k*}}/2$, where $X_{A_{j},A_{j*},B_{k},B_{k*}}$ is the number of observed $A_{j}A_{j*}B_{k}B_{k*}$ genotypes. The estimated observed frequency of *A_j_B_k_* gametes summed from both intra- and intergametic loci is as follows:

$$p\left(A_{j}B_{k}\right)=\left[2X_{A_{j},A_{j},B_{k},B_{k}}+X_{A_{j},A_{j},B_{k},B_{k*}}+X_{A_{j},A_{j*},B_{k},B_{k}}+X_{A_{j},A_{j*},B_{k},B_{k*}}/2\right]/G$$

with *X* being the count of each genotype and *G* is the total number of gametes (Schaid 2004).

**Table 1:** Count of *A_j_B_k_* pairs within genotypes created from parental gametes at locus *A* and *B*, where j* (or k*) is not allele *j* (or *k*)

		Female Gametes			
		*A_j_B_k_*	A_j_B_k*_	A_j*_B_k_	A_j*_B_k*_
Male Gametes	*A_j_B_k_*	2	1	1	1
	A_j_B_k*_	1	0	1#	0
	A_j*_B_k_	1	1#	0	0
	A_j*_B_k*_	1	0	0	0

The ‘#’ indicates where *A_j_B_k_* combinations occur in genotypes but not gametes.

Under the assumption of unlinked and neutral loci, effective population size was estimated using linkage disequilibrium by correcting second-order terms for sampling error:

$$\hat{N}e=\frac{1/3+\sqrt{1/9+2.76{\hat{r}}^{2}\prime }}{2{\hat{r}}^{2}\prime }$$ (1)

where ${\hat{r}}^{2}\prime ={\hat{r}}^{2}-E\left({\hat{r}}_{sample}^{2}\right)$, given ${\hat{r}}^{2}$ is the observed *r*-squared component calculated as the mean ${\hat{r}}_{A_{j}B_{k}}^{2}$ between all alleles over *L*(*L*-1)/2 pairwise comparisons of *L* loci, and $E\left({\hat{r}}_{sample}^{2}\right)=\left[\frac{1}{S}+\frac{3.19}{S^{2}}\right]$ is the term correcting upward bias due to sampling *S* individuals (Waples 2006). The derivation of these equations was the subject of an entire article (Waples 2006); in summary $\hat{N}e$ is a quadratic solution (equation 1) for *Ne* formed by equating ${\hat{r}}^{2}\prime$ to $\frac{1}{3Ne}-\frac{0.69}{Ne^{2}}$, where $\frac{1}{3Ne}$ is the drift term assuming loci are unlinked in a random mating population and$-\frac{0.69}{Ne^{2}}$ is a second-order correction determined by Waples (2006) using simulations.

Large undefined *Ne* estimates occur when the correction due to finite sample size ${\hat{r}}_{sample}^{2}$ is greater than ${\hat{r}}^{2}$, resulting in a negative *Ne* estimate. Negative estimates are plausible and indicate that the sample size *S* is too small, with the correction for sample size being larger than the ${\hat{r}}^{2}$ value determined from the data. *Ne* estimates were determined using program LDNE, where the lower 95% confidence intervals of $\hat{N}e$ were determined by the jackknife method (Waples and Do 2008).

Built into the program of Waples and Do (2008) is a threshold called *P*_crit_, which is used to exclude ${\hat{r}}_{A_{j}B_{k}}^{2}$ from the average ${\hat{r}}^{2}$ if ${\hat{p}}_{A_{j}}$ or ${\hat{p}}_{B_{k}}$ are below the *P*_crit_ threshold. Allele frequencies close to zero can bias ${\hat{r}}_{A_{j}B_{k}}^{2}$ (Waples 2006). We investigate $\hat{N}e$ across a range of *P*_crit_ values because low-frequency alleles are more common in large datasets. Although the theory of Waples (2006) was tested using diallelic loci, it applies equally well in polymorophic data sets (Waples and Do 2010).

### Collection of empirical data

Effective population size was estimated from genotypes of *S. commerson* individuals collected from a defined area, largely within 500 km northwest of Darwin, Northern Territory. Detailed genotyping methods are provided (Supporting Information, File S1).

### Simulations with different effective population sizes

Ten-thousand replicate linkage disequilibrium *Ne* estimates were determined each for a range of population sizes *N* from 3000 to 60,000. The genotypes in each simulated population were generated using program SHAZA (http://molecularfisherieslaboratory.com.au/download-software/) (Macbeth *et al.* 2011). This program simulated *N* first-generation diploid genotypes by random sampling alleles within loci from the empirical allele frequencies of *S. commerson* from the Darwin population. The first *N*/2 genotypes were defined as females and the remainder males. Each individual in the next generation was simulated by random selection of a male and female with replacement. For each parental genotype and for all seven loci a single allele was randomly selected to create an individual diploid genotype. After this process, a total number of *N* individuals was created in four discrete generations.

In this design *N* is approximately equal to *Ne* (Waples 2006). In each replicate, *Ne* was estimated from 5413 generation four genotypes using a plan 2 sampling procedure (Waples 1989). Generation four was used to estimate *Ne* because this was sufficient for ${\hat{r}}^{2}$ to approach an asymptotic value (Sved 1971, Waples 2006). For example, the expectation of ${\hat{r}}^{2}$ in the first generation of simulated genotypes will be zero, resulting in upwardly biased estimates of *Ne*. Simulated genotypes have no missing loci; therefore, before estimating *Ne*, we introduced missing loci to emulate the empirical data structure that had missing loci. The missing loci were introduced for each and every genotype in the simulated data by randomly drawing with replacement a genotype in the empirical data and deleting all loci in the simulated genotype that were found to be missing in the empirical genotype sampled.

### Ne estimates from empirical data with outlier genotypes removed

Putative “outlier” genotypes, defined as genotypes not originating from the focal population under investigation, were identified and removed from the empirical data using a correspondence analysis (CA). The CA algorithm used here was developed in a pilot study by visual assessment of simulated outliers from plots of the first two principal components of a singular value decomposition. Up to 10 CA iterations were performed with iterations continuing until no further outliers are found. In each iteration, outlier genotypes were defined when principal components *PC1* and *PC2* (Appendix A) satisfied a threshold $\sqrt{\left(PC1+PC2\right)}>2$, which removed outliers furthest from the central cluster.

### Ne estimates from empirical data with outlier genotypes removed and genotypes from nontarget species added

To test the sensitivity of *Ne* estimates in genotype samples containing nontarget species, a test was conducted by adding 100 genotypes of a nontarget species (gray mackerel, *Scomberomorus semifasciatus*) to the “cleaned” *S. commerson* data. We would anticipate that adding foreign genotypes will increase $\hat{N}e$ bias and indirectly show that cleaning the data could reduce bias in empirical data estimates. *Scomberomorus semifasciatus* genotypes amplified at five of the seven *S. commerson* loci with alleles at loci SCA47 and SCA49 marked as missing.

### Simulation of genetically divergent populations

To further test the efficiency of the CA algorithm for detecting outlier genotypes, we considered 10 simulated populations that diverged from a founding population across numerous generations. The allele frequencies of the founding population matched those from empirical *S. commerson* samples. Population size was set at *N* = 10,000 and after 100, 200, 500, 1000, or 2000 generations, the population was sampled (sample size of 5413 genotypes). As described previously, the program SHAZA was used to generate *N* genotypes of the founding population. This was followed by creating *N* genotypes each successive generation from random sampling of parental alleles as described previously using an equal sex ratio. Pairwise *F_ST_* values were determined between divergent, simulated populations using Genetix 4.05 software (Belkhir *et al.* 1996−2004). For each of the 10 populations, 100 samples were randomly removed and replaced by 100 random genotypes selected from one of the other nine populations. Following this procedure, we had *n* = 90 populations with 100 immigrant genotypes from nontarget populations and *n* = 10 populations with no immigrants. *Ne* was estimated before and after the data were cleaned using CA.

The ability of the CA algorithm to identify immigrants was compared with the Bayesian clustering approach of STRUCTURE, version 2.3.3 (Pritchard *et al.* 2000). STRUCTURE analysis was applied to the 90 populations that contained 100 immigrants after diverging 2000 generations. Runs were performed by specifying: *k* = 2 clusters, an admixture ancestry model with allele frequencies correlated and a burn in length of 100,000 iterations followed by 100,000 MCMC iterations. One sample location was assumed with no location prior possible.

## Results

### Empirical data

The majority of the 5413 *S. commerson* samples were genotyped with all seven loci (71%), but some samples were genotyped with either six (12%), five (10%), and four (7%) polymorphic microsatellite loci. The numbers of alleles per microsatellite locus varied from 24 (90RTE) to 38 (SCA8), with 65% of alleles across all loci having frequencies less than or equal to 0.01 (Table 2). These low-frequency alleles were selectively removed from data used to estimate *Ne* by the LDNe software depending on the chosen *P*_crit_ thresholds (for more details see: Supplementary genotype results).

**Table 2:** Locus and allele frequency summary

				Number of Alleles With Frequencies		
Locus	*S_L_*	*Na*	Maximum Allele Frequency	Greater Than 0.10	Between 0.01 and 0.001	Less Than 0.001
SCA30	5210	36	0.178	2	17	8
SM3	5206	32	0.183	4	8	13
SM37	4611	37	0.127	2	16	9
SCA47	4781	27	0.486	3	4	14
SCA49	4829	25	0.248	5	5	8
90RTE	5266	24	0.735	1	6	11
SCA8	5139	38	0.216	4	12	11

Sample size at each locus (*S_L_*) and number of alleles (*Na*) for microsatellite loci used to genotype *S. commerson* with the maximum frequency and number of alleles within loci having frequencies less than or greater than the range shown.

Against expectations, LDNE estimates $\left(\hat{N}e\right)$ from empirical data varied systematically across *P*_crit_ values (Table 3). As the *P*_crit_ threshold decreased in magnitude, so too did the magnitude of non-negative estimates of *Ne*. This covariance raised doubts about setting *P*_crit_ to 1/(2*S*) = 1/(2 × 5413)~0.0001, where all singleton alleles would be removed, and the general effectiveness of removing low-frequency alleles for the estimation of *Ne*. The lower confidence interval of $\hat{N}e$ was more stable than the mean estimates but still varied widely from 406 to 24,728 and as such provided no informative value of the lower bound of $\hat{N}e$.

**Table 3:** Estimates of LDNE effective population size $\left(\hat{N}e\right)$ in *S. commerson*

	*P*_crit_						
	0.05	0.02	0.01	0.001	0.0005	0.0001	0.0000
$\hat{N}e$	−40,163*^a^*	−799,447	79,842	17,503	3584	503	418
$\hat{N}e_{lower}$	19,595	24,728	22,209	12,759	3290	489	406
$\hat{N}e_{upper}$	Infinite	Infinite	Infinite	27,158	3921	517	428

**$\hat{N}e$** at different *P*_crit_ thresholds with the upper and lower 95% confidence intervals.

a Negative $\hat{N}e$ estimates indicate a large undefined *Ne*.

### Simulations with different effective population sizes

**Figure 1:**
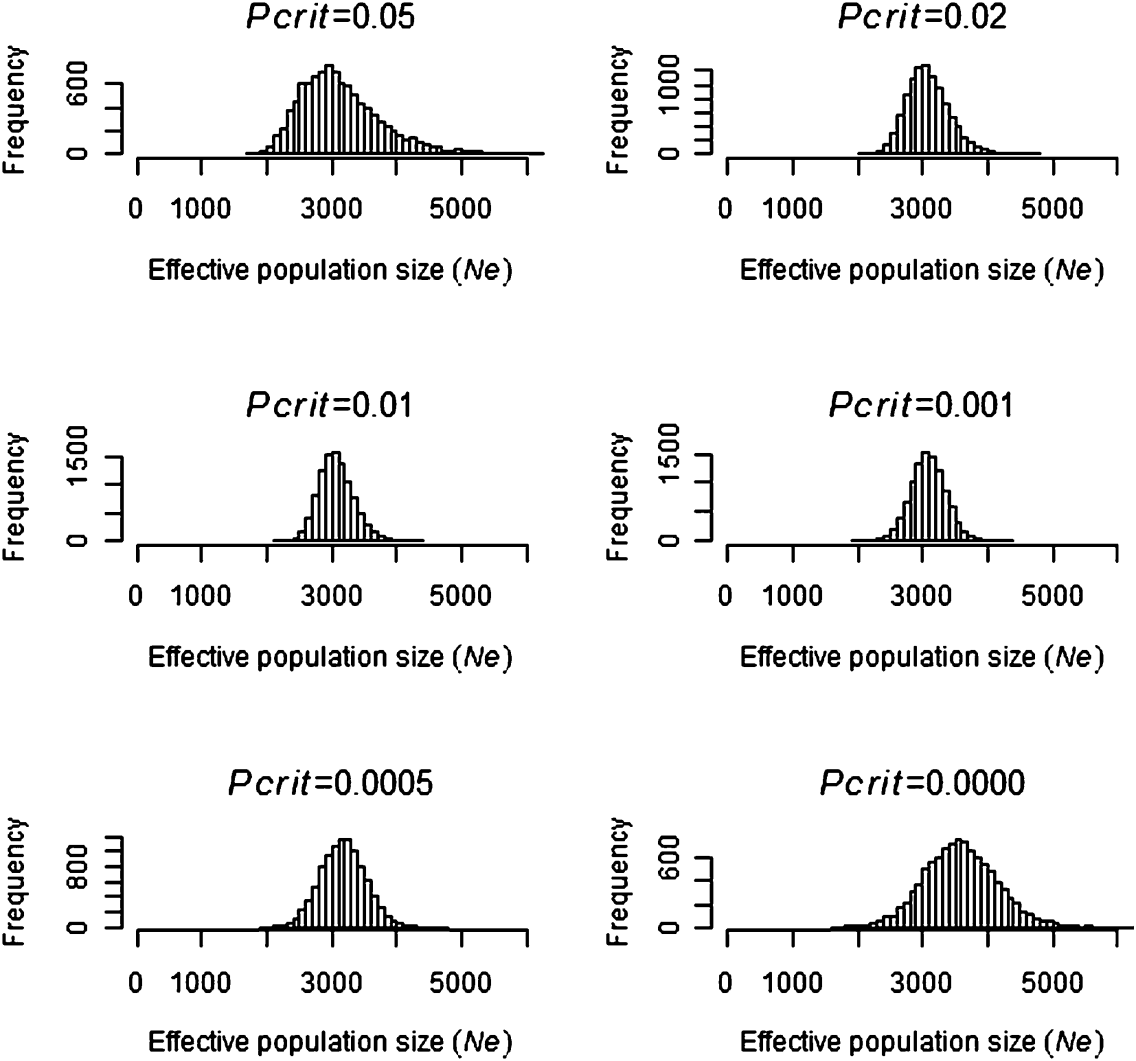
Frequency of 10,000 *Ne* estimates when simulating a population size of *N* = 3000 at different *P*_crit_ values.

**Figure 2:**
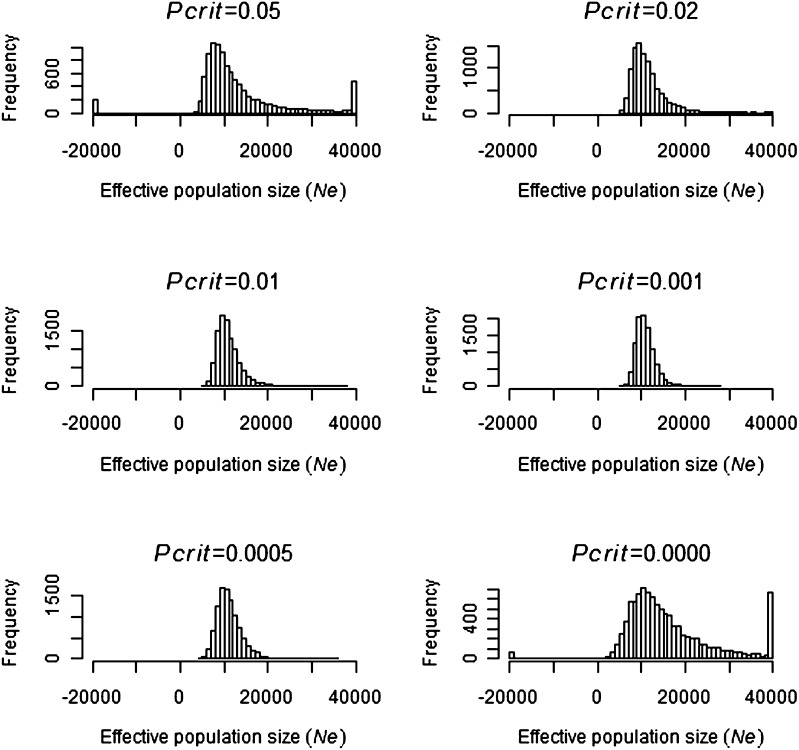
Frequency of 10,000 *Ne* estimates when simulating a population size of *N* = 10,000 at different *P*_crit_ values. The frequency of all *Ne* estimates less than 20,000 and greater than 40,000 were pooled and are indicated on the *x*-axis limits of each graph.

Simulations indicated that 5413 genotype samples should be sufficient to estimate effective population size if the true size was 3000 and 10,000 (Figure 1 and Figure 2). Simulations with *N* = 3000 (Figure 1) had no extreme estimates of *Ne*, whereas simulations with *N* = 10,000 (Figure 2) had a small number of outlier estimates that were greater than 40,000 or less than minus 20,000. In Figures 1 and 2, *P*_crit_ values between 0.01 and 0.001 gave the smallest SD of $\hat{N}e$, illustrating the importance of removing the majority of low frequency alleles.

As expected, simulations with *N* = 100 and *N* = 1000 (Figure S1 and Figure S2) gave more precise estimates of *Ne* than with *N* = 3000 (Figure 1). Increasing *N* from 10,000 to 30,000 and 60,000 (Figure 2; Figure S3, and Figure S4) resulted in a lower precision of $\hat{N}e$ with a greater number of negative and extremely large estimates of *Ne*. An interesting finding was that, at large *N* values such as 60,000, the lower 95% confidence interval (Figure S5) was more precise than the expected mean value (Figure S4), particularly at *P*_crit_ values around 0.01. The results indicate that we had sufficiency in the data to detect the lower 95% confidence interval if *N* was equal to 60000 with the mean lower confidence interval being 22,188 (*P*_crit_ = 0.01).

It is important to note that the smallest 1% of $\hat{N}e$ using *P*_crit_ = 0.0000 determined from the 100th-ranked positive value was 4134, 5308, and 5846 when *N* was 10,000, 30,000, and 60,000, respectively, which revealed an anomaly between the simulation results and empirical data estimates of $\hat{N}e$. If the true *Ne* was larger than 10,000, then the smallest $\hat{N}e$ estimate expected at *P*_crit_ = 0.0000 would be greater than 4134 (*P* < 0.01), which differs from the empirical estimate of 418. Conversely, if the true *Ne* was smaller than or equal to 10,000, then simulations indicated that no negative estimates of $\hat{N}e$ would be expected at *P*_crit_ = 0.02 (*P* < 0.0001), which was contrary to that observed from empirical data with $\hat{N}e=-799447$ (Table 3). This finding highlighted that there was a significant difference between the empirical and simulated data, which was subsequently investigated by examining outlier genotypes.

### *Ne* estimates from empirical data with outlier genotypes removed

The removal of putative outlier genotypes from empirical *S. commerson* data took nine *CA* iterations before there were no genotypes exceeding the $\sqrt{\left(PC1+PC2\right)}>2$ threshold (Figure S6). An order of magnitude increase in $\hat{N}e$ (Table 4) was observed after the first iteration, which removed just 33 outliers (0.6% of total number of genotypes). This finding indicated that putative outlier genotypes can significantly bias *Ne* estimates in empirical data.

**Table 4:** Estimates of *Ne* in *S. commerson* after CA iterations

	*P*_crit_						
CA Iteration (Removed)	0.05	0.02	0.01	0.001	0.0005	0.0001	0.0000
0 (0)	−40,163*^a^*	−799,447	79,842	17,503	3584	503	418
1 (33)	−32,062	−117,650	90,318	112,421	55,074	4968	5051
2 (38)	−33,926	−114,426	91,549	104,569	53,546	8082	7947
3 (51)	−34,571	−104,127	93,996	105,937	48,611	8838	9495
4 (60)	−37,447	−99,305	86,818	113,630	51,105	133,636	171,370
5 (90)	−38,487	−86,051	89,982	302,878	−448,815	−51,226	−36,471
6 (119)	−35,678	−76,242	120,453	302,946	−146,528	−38,189	−30,685
7 (153)	−38,909	−75,672	101,714	610,512	−69,972	−16,082	−16,082
8 (170)	−32,038	−65,015	296,541	−795,394	−58,191	−14,132	−14,132
9 (174)	−32,371	−67,105	550,582	−420,513	−48,637	−14,059	−14,059

The removal of putative outliers from nine sequential CA iterations with the cumulative number of genotypes removed indicated in brackets and the following estimates of *Ne* at different *P*_crit_ thresholds. CA, correspondence analysis.

a Negative $\hat{N}e$ estimates indicate a large undefined *Ne*.

After the nine *CA* iterations, 3.2% of samples were removed. Subsequent *Ne* estimates on the cleaned data were negative at all *P*_crit_ thresholds, except when *P*_crit_ was 0.01 $\left(\hat{N}e=550582\right)$. This finding indicated that a *P*_crit_ of 0.01 provided the greatest accuracy, as it had the smallest confidence interval assessed by the fact that it was the only *P*_crit_ value in which the correlation of alleles between loci was greater than that expected from sampling error. At this *P*_crit_ value $\hat{N}e$ was relatively stable at around 80,000 to 100,000 until the last two iterations with $\hat{N}e$ increasing to 550,582. When *P*_crit_ was 0.01, the harmonic mean of $\hat{N}e$ across all nine iterations was 110,000.

The lower 95% confidence interval of the *Ne* estimates $\left(\hat{N}e_{lower}\right)$ from Table 4 is reported in Table 5. The lower confidence intervals appeared to be more stable than the estimates provided in Table 4 when the *P*_crit_ values were equal to or greater than 0.001. The range of $\hat{N}e_{lower}$ estimates when *P*_crit_ = 0.01 were within 21% of each other with a harmonic mean of 24000.

**Table 5:** Lower 95% confidence interval of *Ne* from *S. commerson* genotypes

	*P*_crit_						
CA Iteration (Removed)	0.05	0.02	0.01	0.001	0.0005	0.0001	0.0000
0 (0)	19,595	24,728	22,209	12,759	3290	489	406
1 (33)	22,540	30,509	22,943	26,461	17,594	1988	2046
2 (38)	21,571	30,713	23,011	26,119	17,498	2849	2913
3 (51)	21,232	31,541	23,144	33,737	25,337	7606	8131
4 (60)	20,110	31,970	22,720	26,879	16,904	16,696	14,799
5 (90)	19,615	33,487	22,809	42,238	60,094	−271,390*^a^*	−83,353
6 (119)	20,379	35,118	24,305	29,804	53,307	−98,902	−59,311
7 (153)	19,174	34,947	23,471	31,098	80,748	−35,452	−35,453
8 (170)	21,646	37,832	27,703	36,446	151,392	−23,066	−23,066
9 (174)	21,445	37,064	28,922	35,858	−615,338	−23,260	−23,260

The removal of putative outliers from nine CA iterations with the cumulative number of genotypes removed indicated in brackets and the following estimates of the lower 95% confidence interval ($\hat{N}e_{lower}$) at different *P*_crit_ thresholds. CA, correspondence analysis.

a Negative $\hat{N}e$ estimates indicate a large undefined Ne.

### *Ne* estimates from empirical data with outlier genotypes removed and genotypes from nontarget species added

Adding nontarget species (gray mackerel, *S. semifasciatus*) to the “cleaned” *S. commerson* data significantly reduced *Ne* estimates (Table 6). Considering the total sample size was 5413, the results clearly show that only a small proportion of nontarget species can have a large impact on linkage disequilibrium estimates of *Ne*. For example, adding as few as eight (0.15%) *S. semifasciatus* genotypes resulted in a 5.7-fold reduction in $\hat{N}e$ when *P*_crit_ = 0.01. All of the 200 nontarget gray mackerel genotypes were identified and removed by the first iteration of CA analysis compared with the nine iterations that were required with the empirical data (Table 4). This finding suggests that the putative outliers in the empirical data were more similar to *S. commerson* than *S. semifasciatus*.

**Table 6:** Effect of *S. commerson Ne* estimates when adding nontarget species

	*P*_crit_						
Gray Mackerel Genotypes Added	0.05	0.02	0.01	0.001	0.0005	0.0001	0.0000
0	−32,371*^a^*	−67,105	550,582	−420,513	−48,637	−14,059	−14,059
1	−32,382	−67,686	566,612	−410,564	−48,310	1303	1303
2	−32,315	−67,583	719,220	−356,551	−47,594	1031	1031
4	−35,620	−70,777	159,027	−966,684	−50,839	1138	1138
8	−36,871	−79,371	95,957	206,370	3930	1179	1179
16	−37,624	−94,247	43,218	2030	1088	1238	1238
32	−45,964	−1,040,355	16,140	1104	985	1233	1233
64	626,218	5420	2896	700	776	974	1014
100	23,439	5946	813	553	654	806	862
200	2189	418	233	376	455	547	620

Starting with *S. commerson* data with 174 outliers removed by nine CA iterations, *Ne* estimates at different *P*_crit_ thresholds were determined after progressive addition of gray mackerel (*S. semifasciatus*) genotypes. CA, correspondence analysis.

a Negative $\hat{N}e$ estimates indicate a large undefined Ne.

Our *S. semifasciatus* samples did not amplify at loci SCA47 and SCA49. Removing all genotypes in the empirical data that did not amplify at these two loci produced a similar $\hat{N}e$ profile to Table 3, indicating that *S. semifasciatus* cannot be solely implicated in the anomaly between the simulated and empirical data.

### Simulation of genetically divergent populations

Ten populations simulated after divergence from a common founder population had average pairwise *F_ST_* values of 0.004, 0.010, 0.027, 0.048, and 0.091 after 100, 200, 500, 1000, and 2000 generations, respectively. With no mixing of the populations during genotype sampling, *Ne* estimates approximated the simulated population size (*N* = 10,000, Table 7).

**Table 7:** Harmonic mean of $\hat{N}e$ before and after outlier genotypes removed

	Before Outlier Genotypes Removed		After Outlier Genotypes Removed	
Generations	No Immigrants, *n* = 10	With Immigrants, *n* = 90	No Immigrants, *n* = 10	With Immigrants, *n* = 90
*P*_crit_ = 0.000				
100	9896	6236	13,911	17,100
200	10,543	3037	11,947	13,973
500	10,029	1282	11,151	11,558
1000	97,734	571	10,548	11,049
2000	11,834	176	12,359	12,295
*P*_crit_ = 0.010				
100	10,732	11,096	10,841	11,267
200	10,557	10,932	10,670	11,094
500	10,211	9420	10,217	10,003
1000	9595	7629	9691	9736
2000	10,407	4456	10,508	10,564

Harmonic mean of $\hat{N}e$ at two *P*_crit_ thresholds in simulated populations with *N* = 10,000 and sample size *S* = 5413 containing no immigrants or with 100 genotypes drawn from a single immigrant population. The immigrants are from populations diverging after a different number of generations from a common population. The harmonic mean in each column was based on *n* separate$\hat{N}e$ estimates before and after outlier genotypes were removed using the CA algorithm. CA, correspondence analysis.

Ninety populations with 100 immigrants were created from pairs of the 10 divergent populations. Across these 90 populations CA analysis found an average (SD) of 7 (4), 18 (8), 44 (12), 74 (12), and 93 (6) immigrants after 100, 200, 500, 1000, and 2000 generations, respectively. The average number of CA iterations required before no more immigrants could be detected were 3.4, 3.6, 3.6, 3.1, 3.0 after 100, 200, 500, 1000, and 2000 generations, respectively. As a comparison, the program STRUCTURE was not able to distinguish the immigrants, even after 2000 generations of divergence. When two populations were specified in STRUCTURE 97 of the 100 immigrants and 47.3% of the remaining 5313 samples were partitioned into the same population. This finding indicated that there was not sufficient genetic divergence between the populations to cluster the small proportion of immigrants into a separate population.

In the presence of 100 immigrants, there was a downward bias in $\hat{N}e$ of the focal population for *P*_crit_ values of 0.00 and 0.01 (Table 7) as the number of generations of divergence increased. After outlier genotypes were removed *Ne* estimates were more consistent with an expected value of *N* = 10,000. After outlier (*i.e.*, immigrant) genotypes were removed by CA, the smallest bias and highest accuracy of *Ne* occurred when *P*_crit_ = 0.01.

## Discussion

Palstra and Ruzzante (2011) urged further theoretical developments to avoid a downward bias in estimating linkage disequilibrium *Ne* in naturally occurring metapopulations. Our results have demonstrated that under certain circumstances even estimates for focal populations can be downwardly biased. We believe this bias could be due to the presence of 1) nontarget species and 2) immigrant genotypes from diverged populations among the samples taken for estimation. Importantly, only a few ‘contaminant’ genotypes can severely bias *Ne* estimates.

The nature of how the contaminant genotypes differ is at the crux of what causes the downward bias in effective population size. We assumed that contaminant genotypes were from transient individuals that did not interbreed among members of the focal population. Our results are therefore not in disagreement with a study in which the authors showed that linkage disequilibrium estimates of effective population size are robust to populations displaying equilibrium migration and mating over many generations (Waples and England 2011). We propose the bias expectations are different for contamination by unrelated species or reproductively isolated subpopulations *vs.* subpopulations from the same metapopulation.

The CA algorithm performed well in identifying and removing nontarget genotypes that were added to simulated population samples. In our hands, standard methods of population clustering such as STRUCTURE (Pritchard *et al.* 2000) were incapable of identifying the simulated immigrants. The threshold value of 2 used in the CA algorithm was developed by trial and error as a reasonable threshold to exclude outlier genotypes without removing too many target population genotypes. A series of scatter plots on principal coordinates is shown after each iteration of removing outliers on the threshold (Figure S6). The pattern in this series was typical for many of the simulations runs in which a final cluster of points becomes clearly visible. As expected, as the *F_ST_* between nontarget and the target populations decreased, it was more difficult to detect the nontarget genotypes using the CA algorithm. Although the simulated results seem sensible, the theoretical basis of this algorithm and its generalizability to removing nontarget genotypes in other data sets would provide additional support for this method. Our findings suggest that it is worthwhile to detect and remove putative nontarget genotypes prior to LDNE analysis.

Our simulated divergent populations were implemented using a simple Wright-Fisher model with mating modified such that gametes were chosen from populations having equal numbers in each sex. This model was used by Waples (2006); however, many other models could have been used, including those with mutation and selection (Der *et al.* 2011). These additional processes would cause a larger divergence at the same number of generations compared with the simple genetic drift model used in our study.

Our investigation suggests that mackerel genotypes collected around Darwin contained a small proportion from genetically divergent *S. commerson* population(s) or from congeneric species. It is possible that tissue samples of closely related species were taken inadvertently, thus mimicking an admixed *S. commerson* population. Our 100 gray mackerel (*S. semifasciatus*) samples amplified at five of the seven loci used in our study, whereas another closely related endemic species (*Scomberomorus queenslandicus*) amplifies at all the seven loci (unpublished data). The fact that all gray mackerel genotypes were successfully removed by our CA method does indicate that our methods work well when nontarget species are implicated. We would expect intermediate results when populations are at varying levels of population divergence as indicated by our simulations.

We assumed no genotyping errors when estimating linkage disequilibrium, although prescreening of the data resulted in one locus being removed due to a deviation from Hardy Weinberg equilibrium. Although this deviation might indicate the presence of a null allele error, there could be other errors, such as allelic dropout errors. Random dropout errors are not expected to change the expectation of linkage disequilibrium estimates nor the outcome of the expected *Ne* estimate.

Assuming that all samples represented *S. commerson*, it is likely that the population adjacent to Darwin is an admixed population containing small numbers of individuals from genetically distinct populations. These individuals could also have been transient vagrants of genetically distinct populations of *S. commerson* (Sulaiman and Ovenden 2010; Fauvelot and Borsa 2011) that were sampled in the same geographical region. The hypothesis that a small (rather than large) number of immigrant genotypes were present in the empirical genotypes is supported by the observations that (1) most adults in a mark-recapture study were found to move less than 100km per year parallel to the shore and (2) isotope signatures in the sagittal otolith carbonate of *S. commerson* indicated spatial separation across northern Australia (Newman *et al.* 2009).

In our *S. commerson* data, it was very difficult to get a precise estimate of $\hat{N}e$. Before “cleaning” the data with CA, *Ne* estimates varied at different *P*_crit_ levels, including some negative estimates of *Ne*. Using a *P*_crit_ value of 0.01 the likely $\hat{N}e$ seems very large with an estimate of 110,000 from empirical data. We believe this estimate to be unreliable as inferred from the lack of sufficiency of the data when estimating the mean *Ne* with *N* = 600,000 (Figure S4).

Negative estimates of *Ne* are counterintuitive and indicate that the true *Ne* is large and undefined. Waples and Do (2010) point out that even if the *Ne* estimate is negative, if adequate data are available, the lower bound of the confidence interval may be finite and can provide useful information. This finding was also supported by our simulations with large *N* values in which the lower 95% confidence interval for *S. commerson* appears to be much more stable than the estimate and upper limits. Using a *P*_crit_ value of 0.01, we found that the lower 95% confidence interval gave a harmonic mean of $\hat{N}e=24000$ from empirical data. More loci could be used to achieve more precise estimates of *Ne*. However, we had sufficiency in the data to detect *Ne* when *N* = 30,000 (Figure S3, *P*_crit_ = 0.01). We also had sufficiency in the data when estimating the lower 95% confidence of *Ne* with *N* = 60,000 giving $\hat{N}e_{lower}=22188$ (Figure S5, *P*_crit_ = 0.01). We conclude from these simulations that the empirical *Ne_lower_* estimate of 24,000 is reasonably reliable. In ecological terms 24,000 represents a large and stable genetic population size, and we would expect to reach a similar conclusion with the addition of more loci.

This study was primarily focused on the bias in the linkage disequilibrium estimation of *Ne* when a population may include genetically divergent conspecifics. There are many other approaches used to estimate *Ne* that have different underlying assumptions (Barker 2011) and that should be evaluated as being suitable for the estimation of *Ne* in large, naturally occurring populations. A natural progression in this area of research is to develop inferences of census population sizes based on effective size estimates (Palstra and Ruzzante 2008) and how these could be used to assist management of natural resource species.

Realistic simulations have showed that it is possible to make effective population size estimates using the linkage disequilibrium method with finite confidence limits up to several thousand depending on the number of loci and genotypes assayed. Estimates of effective size made from samples taken from naturally occurring populations must be treated with caution. We recommend pretreatment of the sampled genotypes to identify outliers, particularly if the population being studied is sympatric with closely related species, or is possibly receiving immigrants from adjacent populations.

## Supplementary Material

Supporting Information

## Appendix A

### CA R script

The genotype file is presented as an incidence matrix *Z* having columns for each allele within every locus. For each and every genotype a single row marking the presence ‘1’ or absence ‘0’ of each allele present within each column is appended to fill the content of the *Z* matrix. The R code modified from Nenadic and Greenacre (2006) converts the incidence matrix to a format that can be read and manipulated by R (R Development Core Team 2011) with the first two principal components *PC1* and *PC2* determined as follows:

Z <- data.matrix(Z)  # convert to matrix
P <- Z / sum(Z)  # proportional contribution
rm <- apply(P, 1, sum)  # sum rows
cm <- apply(P, 2, sum)  # sum columns
eP <- rm %*% t(cm)  # multiply by transpose
dec <- svd((P - eP) / sqrt(eP))  # singular value decomposition
PC1 <- dec$u[,1] * dec$d[1] / sqrt(rm)  # Principal component 1
PC2 <- dec$u[,2] * dec$d[2] / sqrt(rm)  # Principal component 2
