# Supplementary material for: Linkage Disequilibrium Estimation of Effective Population Size with Immigrants from Divergent Populations: A Case Study on Spanish Mackerel (Scomberomorus commerson)
Source: G3 (Bethesda). 2013 Apr 1;3(4):709–17. doi: 10.1534/g3.112.005124 (PMC3618357; doi:10.1534/g3.112.005124)
Supplement: Supporting Information [file supp_3_4_709__index.html]

Linkage Disequilibrium Estimation of Effective Population Size with Immigrants from Divergent Populations: A Case Study on Spanish Mackerel (Scomberomorus commerson) — Supporting Information 

# Linkage Disequilibrium Estimation of Effective Population Size with Immigrants from Divergent Populations: A Case Study on Spanish Mackerel (*Scomberomorus commerson*)

## Supporting Information for Macbeth *et al.*, 2013

**Files in this Data Supplement:**

- Supporting Information - Figures S1-S7 and File S1 (PDF, 762 KB)
- Figure S1 - Frequency of 10000 *Ne* estimates when simulating a population size of *N*=100 at different *P*crit values (PDF, 155 KB)
- Figure S2 - Frequency of 10000 *Ne* estimates when simulating a population size of *N*=1000 at different *P*crit values (PDF, 158 KB)
- Figure S3 - Frequency of 10000 *Ne* estimates when simulating a population size of *N*=30000 at different *P*crit values (PDF, 157 KB)
- Figure S4 - Frequency of 10000 *Ne* estimates when simulating a population size of *N*=60000 at different *P*crit values (PDF, 161 KB)
- Figure S5 - Frequency of lower 95% confidence interval of *N^e* from 10000 estimates when simulating a population size of *N*=60000 at different *P*crit values
- Figure S6 - Correspondence analysis plots... (PDF, 114 KB)
- Figure S7 - Graphical output from Microchecker software showing observed (X) and expected (red vertical bars) frequency of homozygotes (left panel) and heterozygotes (right panel) for 500 genotypes from 2004 collected adjacent to Darwin (PDF, 149 KB)
- File S1 - Supplemental Data (PDF, 78 KB)
