# Supplementary material for: Linkage Disequilibrium Estimation of Effective Population Size with Immigrants from Divergent Populations: A Case Study on Spanish Mackerel (Scomberomorus commerson)
Source: G3 (Bethesda). 2013 Apr 1;3(4):709–17. doi: 10.1534/g3.112.005124 (PMC3618357; doi:10.1534/g3.112.005124)
Supplement: Supporting Information [file supp_g3.112.005124_005124SI.pdf]

G3·Genes | Genomes | Genetics

Linkage disequilibrium estimation of effective population size with immigrants from divergent populations: a case study on Spanish mackerel (*Scomberomorus commerson*).

Gilbert Michael Macbeth[*] , Damien Broderick[*], Rik C Buckworth[§] Jennifer R Ovenden[*]

[*]Molecular Fisheries Laboratory, Queensland Government, PO Box 6097, St Lucia, Queensland, 4072, Australia.
[§] CSIRO Marine & Atmospheric Research, Wealth from Oceans National Research Flagship, GPO Box 2583, Brisbane, Queensland, 4001, Australia

Corresponding author:
Current address: Centre for Applications in Natural Resource Mathematics, School of Mathematics and Physics, The University of Queensland, Queensland, 4072 Australia.
Phone: +61 7 33466518
Fax: +61 7 33466501
Email: macbeth@bigpond.net.au

DOI: 10.1534/g3.112.005124

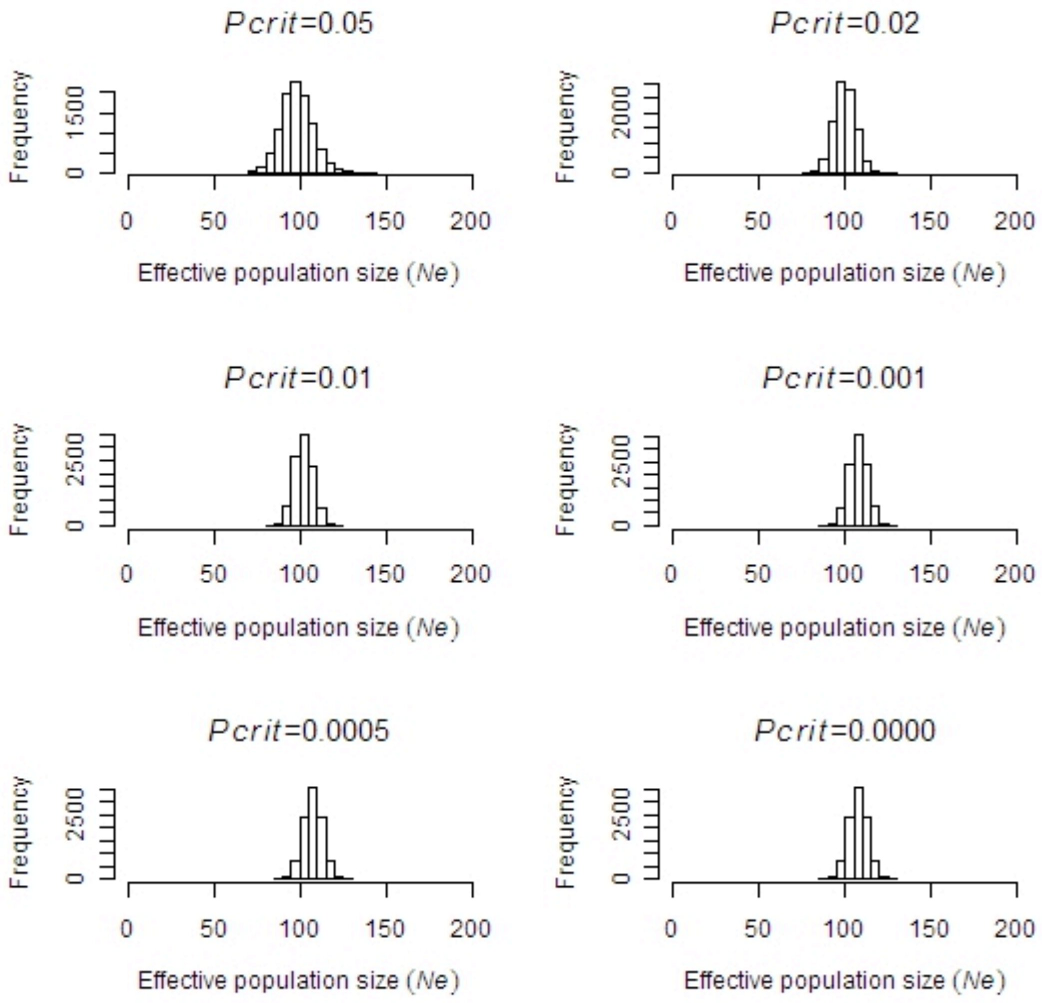

Figure S1 Frequency of 10000 *Ne* estimates when simulating a population size of *N*=100 at different P_{crit} values.

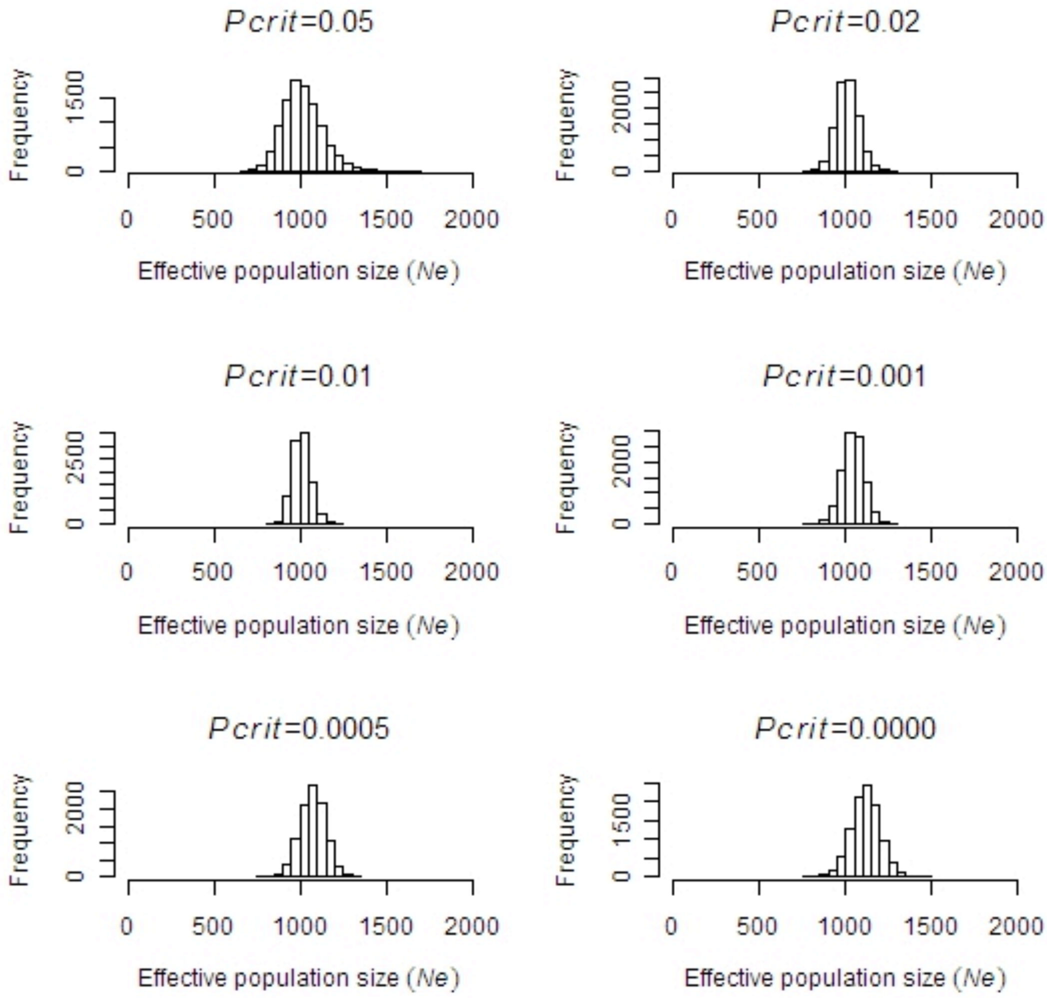

Figure S2 Frequency of 10000 *Ne* estimates when simulating a population size of *N*=1000 at different P_{crit} values.

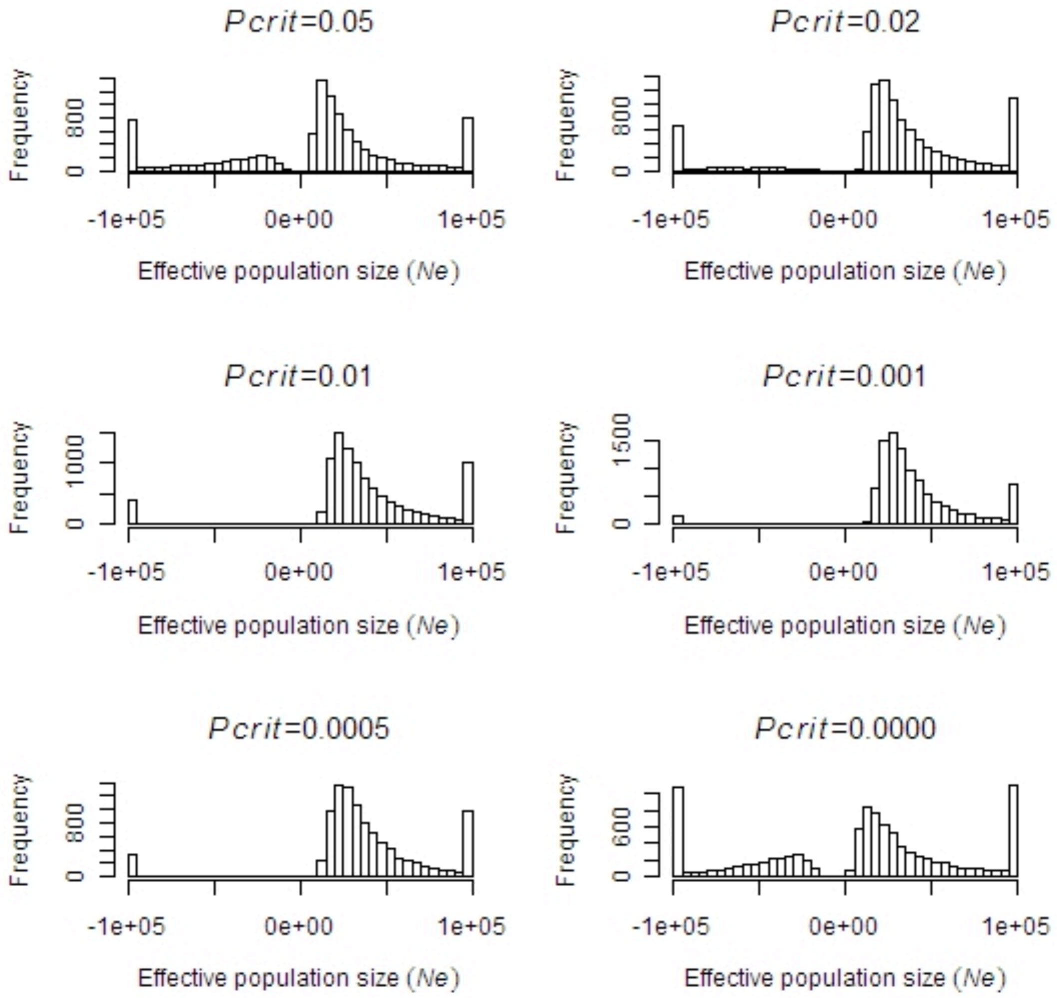

Figure S3 Frequency of 10000 *Ne* estimates when simulating a population size of *N*=30000 at different P_{crit} values. The frequency of all *Ne* estimates less than 100000 and greater than 100000 were pooled and are indicated on the *x*-axis limits of each graph.

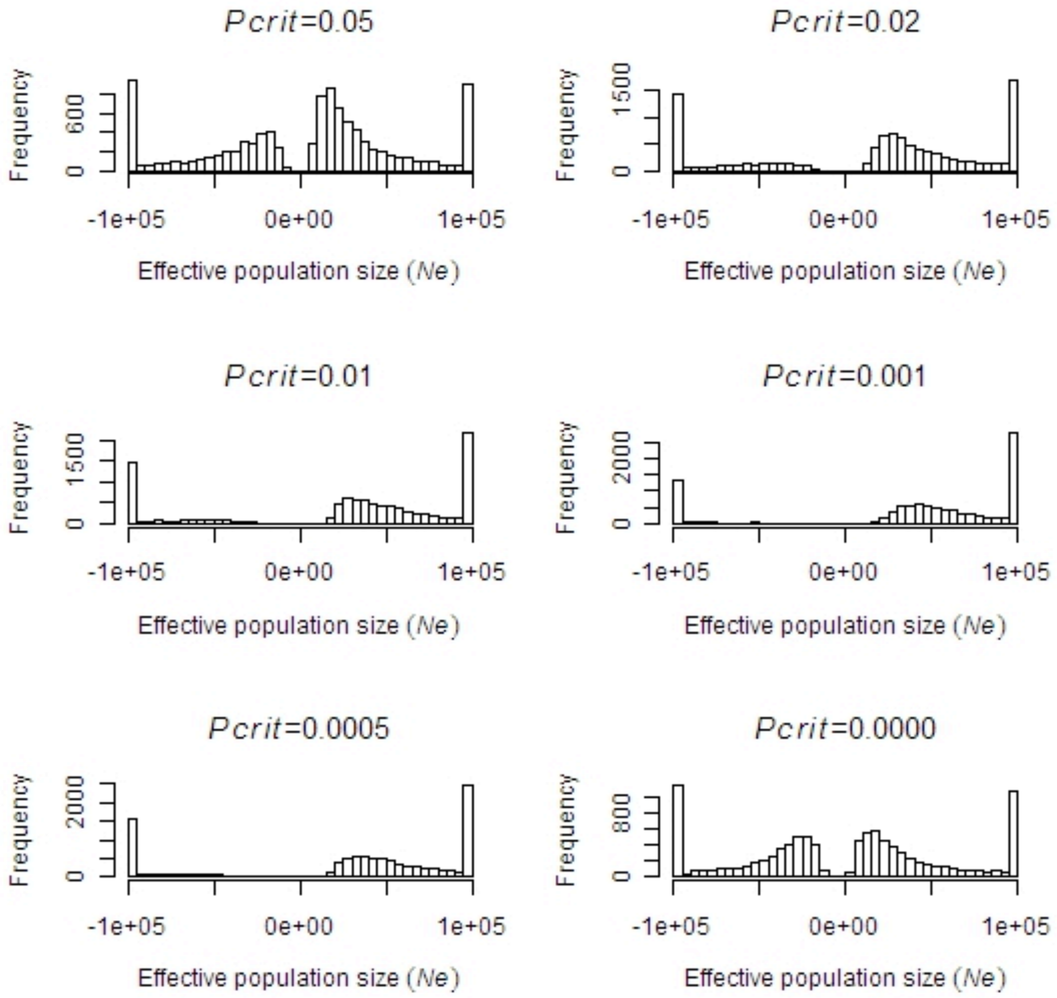

Figure S4 Frequency of 10000 *Ne* estimates when simulating a population size of *N*=60000 at different P_{crit} values. The frequency of all *Ne* estimates less than 100000 and greater than 100000 were pooled and are indicated on the *x*-axis limits of each graph.

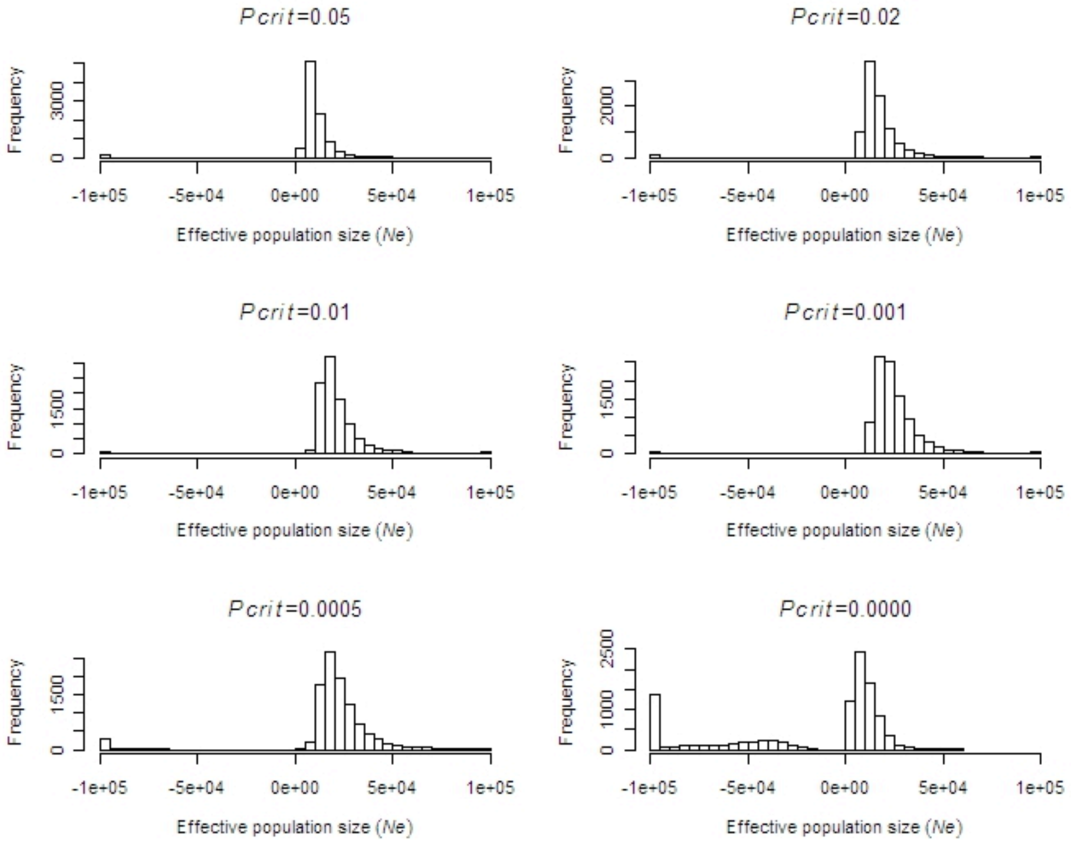

Figure S5 Frequency of lower 95% confidence interval of $\hat{N}e$ from 10000 estimates when simulating a population size of *N*=60000 at different P_{crit} values. The frequency of all *Ne* estimates less than 100000 and greater than 100000 were pooled and are indicated on the *x*-axis limits of each graph.

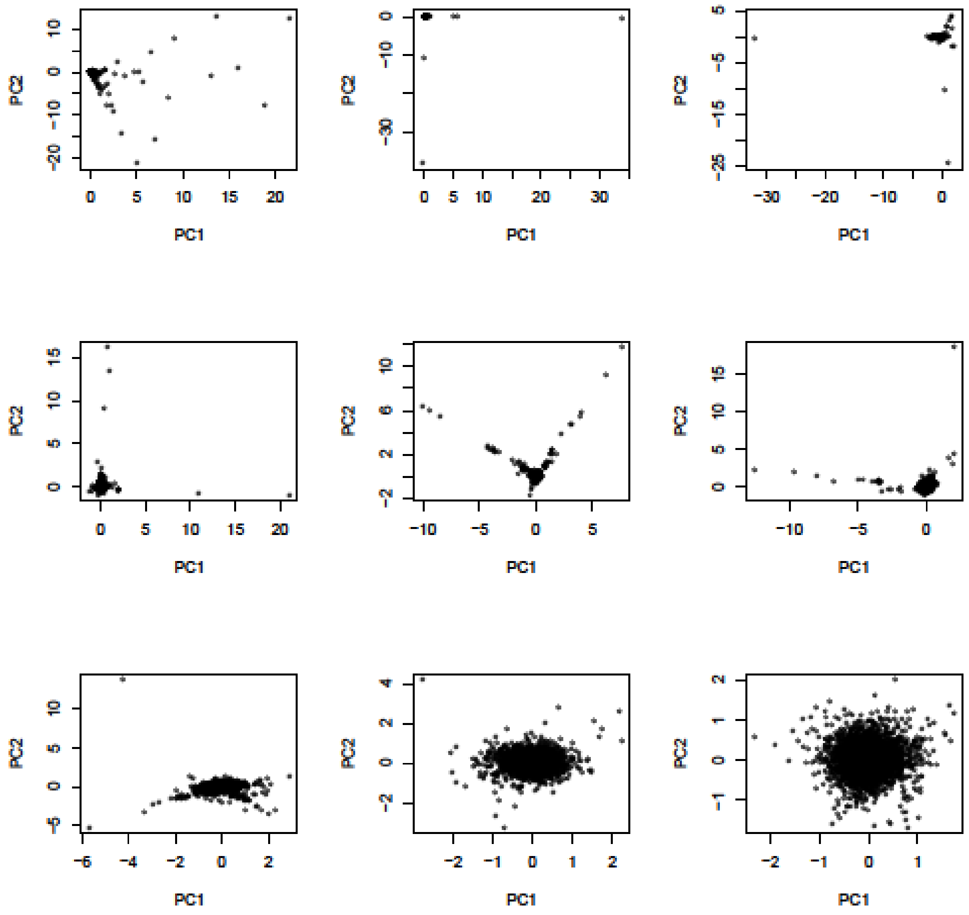

Figure S6 Correspondence analysis plots after nine iterations of removing outliers in the empirical mackerel data that satisfied the threshold $\sqrt{(PC1 + PC2)} > 2$ where *PC1* and *PC2* are the first and second principal components. Iterative steps are from top left to right moving down rows. The last plot 8 shows a cluster ball of genotypes after removing 116 genotypes from 5413 genotypes. One more iteration (not shown) removed 4 additional points.

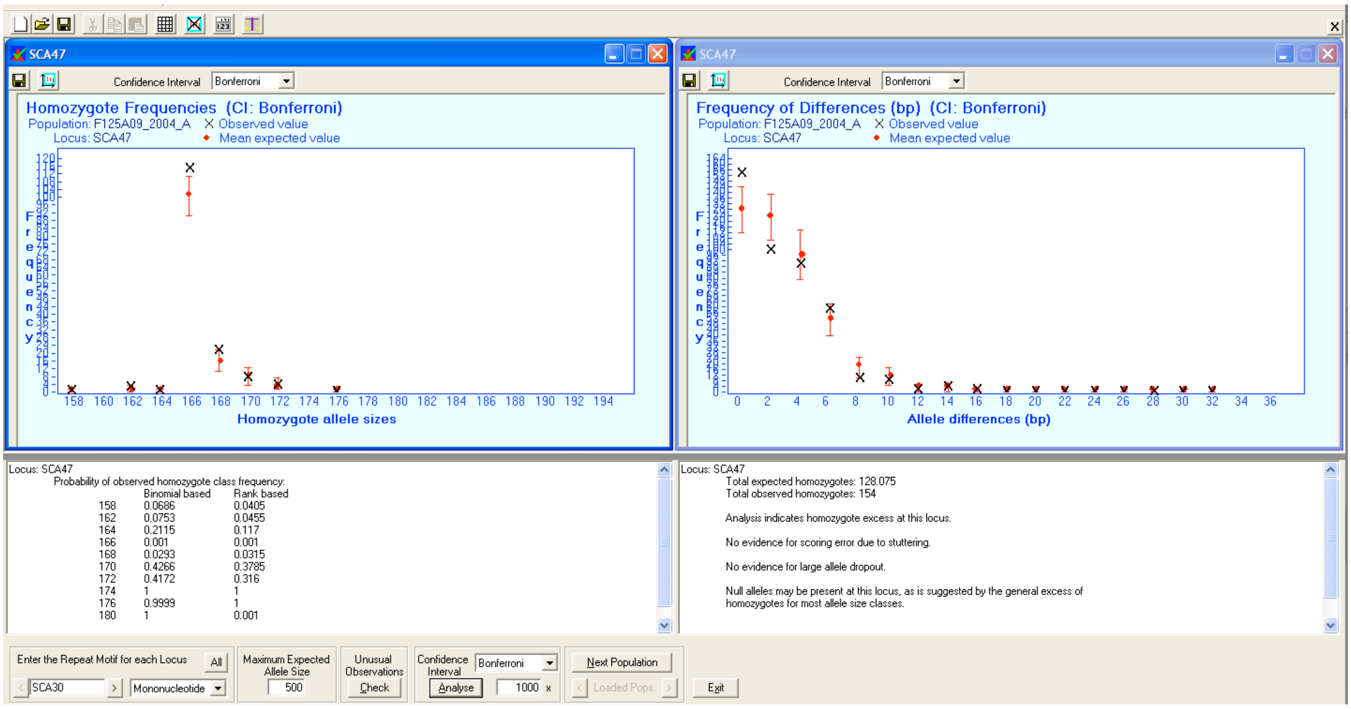

Figure S7 Graphical output from Microchecker software showing observed (X) and expected (red vertical bars) frequency of homozygotes (left panel) and heterozygotes (right panel) for 500 genotypes from 2004 collected adjacent to Darwin.

File S1
Supplemental Data

Supplementary genotype methods

Tissue samples were taken from fish and stored in 90% ethanol or a saturated $NaCl_2$ solution containing 20% dimethyl sulphate. In total, 5413 genotypes from seven polymorphic microsatellite loci were collected between 2003 and 2006.

Samples were genotyped with seven di-nucleotide microsatellite loci; *90RTE* (Van Herwerden *et al.*, 2000), *SCA8, SCA30, SCA47, SCA49* (Gold *et al.*, 2002), *SM3* (GenBank AY700810.1) and *SM37* (GenBank AY700844.1). Genomic DNA was extracted using the salting-out method (Sambrook *et al*., 1989). Microsatellite amplifications for the seven loci were performed in four multiplexed reactions in 96-well plates using Perkin Elmer (Waltham, MA, U.S.A.) 9600 and 9700 series thermocyclers. The PCR volume per well was six microliters with QIAGEN® (Hilden, Germany) master mix (containing Taq polymerase and magnesium chloride) and QIAGEN® (Hilden, Germany) Q-solution was used to facilitate multiplexing. Mineral oil was used to control evaporation during cycling. Cycling conditions consisted of denaturation at 95°C for 15 min, followed by 37 cycles of 94°C for 30 sec at 56°C for 45 sec and 72°C for 1 min 30 sec. A final extension at 72°C for 45 min was used to ensure complete addition of adenine to the PCR product. Microsatellite gel separation and scoring was performed on a Life Technologies™ (Carlsbad, CA, U.S.A.) ABI™ 3130*xl* Genetic Analyser. Life Technologies™ Genemapper™ 3.7 software was used to score alleles, to assign them to bin classes and export genotype information for subsequent analyses.

Empirical data was tested for deviations from Hardy-Weinberg equilibrium (HWE) and linkage disequilibrium using Genepop-on-the-web v4.0.10 (Rousset, 2008). For HWE tests, all locus *x* population combinations were tested. Tests for linkage disequilibrium considered all combinations of locus pairs for each population. Tests were made with successively larger batch sizes in Genepop until a stable result was obtained. Bonferroni corrections for simultaneous tests were applied commencing with an a level of 0.05. The software Microchecker (Van Oosterhout *et al.*, 2004) was used to examine cases of deviation for Hardy-Weinberg equilibrium for microsatellite data. Microsatellite data was analysed in blocks of less than 500 samples to avoid the upper limit of the Microchecker software.

Supplementary Simulation Results

Figures S1 to S6 are referred to within the main manuscript. Briefly the frequency distribution of *Ne* estimates is a good indicator of the precision obtained from the seven polymorphic loci used in this study. Ideally

a tight cluster of *Ne* estimates is desirable (Figure S1, *Pcrit*=0.01). When there is insufficient genotype data negative and or very large estimates can occur (Figure S4). The lower 95% confidence interval of *Ne* (Figure S5) was less variable than the mean expectation (Figure S5).

Supplementary genotype results

Average observed heterozygosity across seven microsatellite loci was 0.762 and the average expected heterozygosity was 0.802. Tests for Hardy-Weinberg equilibrium rejected the null hypothesis for all seven loci. Locus-by-locus analysis with Microchecker showed that for some alleles there was a difference in the observed and expected number of homozygotes, inferring null alleles may be present. Nulls were predicted by the software at loci *Sca49* and *Sca47* at frequencies ranging from 0.03 to 0.09, and nulls were detected at lower frequencies at some other loci. Graphical representation by Microchecker of the observed and expected frequency of heterozygotes, plotted against the number of base pairs separating the two alleles, revealed a deficit in heterozygotes when alleles were separated by two base pairs and a compensatory increase in the observed number of homozygotes (Figure S7). This could be explained by a slight scoring error, which may have been responsible for the null allele predictions made by Microchecker and which may have been compounded by large sample sizes in the HWE tests. Wakefield (2010) confirms that rejection of the null hypothesis using conventional *p*-values is more likely when sample sizes are large and recommends a Bayesian framework in these cases. Thus, a small proportion of heterozygote genotypes were under-represented in the microsatellite data. There was unlikely to be cause a systematic bias in the microsatellite data, as the controlling factor in their omission was similarity in allele size, which should occur evenly across alleles independent of their frequency or size, and across samples independent of biological factors.

References

Gold, J. R., E. Pak, and D. A. DeVries, 2002 Population structure of king mackerel (*Scomberomorus cavalla*) around peninsular Florida, as revealed by microsatellite DNA. Fisheries Bulletin 100:491-509.

Rousset, F. 2008 Genepop 007: a complete re-implementation of the genepop software for Windows and Linux. Molecular Ecology Resources 8:103-106.

Sambrook, J., E. F. Fritsch, and T. Maniatis, 1989 Molecular Cloning: A Laboratory Manual, 2nd edn. Cold Spring Harbour Laboratory Press, Cold Spring, New York.

Van Herwerden, L., J. Benzie, L. Peplow, and C. Davies, 2000 Microsatellite markers for coral trout (*Plectropomus laevis*) and red throat emperor (*Lethrinus miniatus*) and their utility in other species of reef fish. Molecular Ecology 9:1919-1952.

Van Oosterhout, C., W. F. Hutchinson, D. P. M. Wills, and P. Shipley, 2004 MICROCHECKER: software for identifying and correcting genotyping errors in microsatellite data. Molecular Ecology Notes 4:535-538.

Wakefield, J. 2010 Bayesian methods for examining Hardy-Weinberg equilibrium. Biometrics 66:257-265.
