# Supplementary material for: Linkage Disequilibrium Estimation of Effective Population Size with Immigrants from Divergent Populations: A Case Study on Spanish Mackerel (Scomberomorus commerson)
Source: G3 (Bethesda). 2013 Apr 1;3(4):709–17. doi: 10.1534/g3.112.005124 (PMC3618357; doi:10.1534/g3.112.005124)
Supplement: Supporting Information [file supp_g3.112.005124_FigureS5.pdf]

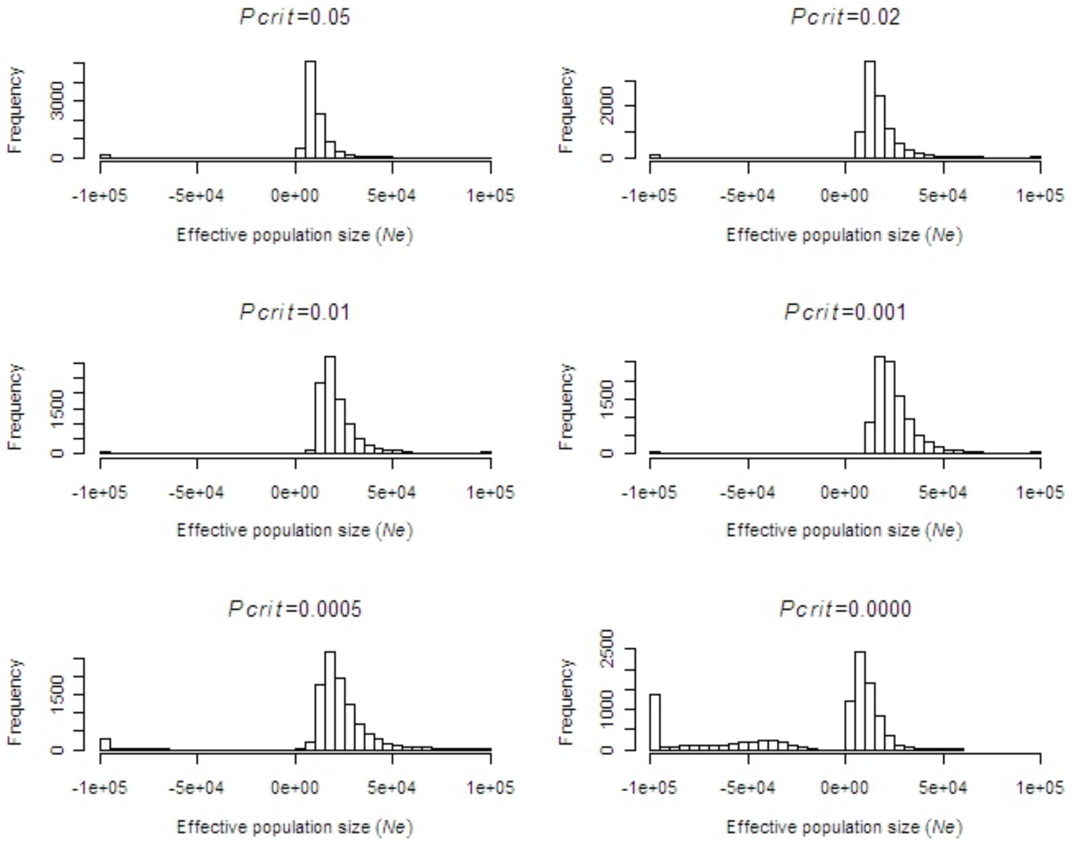

Figure S5 Frequency of lower 95% confidence interval of $\hat{N}e$ from 10000 estimates when simulating a population size of *N*=60000 at different P_{crit} values. The frequency of all *Ne* estimates less than 100000 and greater than 100000 were pooled and are indicated on the *x*-axis limits of each graph.
