# Supplementary material for: Linkage Disequilibrium Estimation of Effective Population Size with Immigrants from Divergent Populations: A Case Study on Spanish Mackerel (Scomberomorus commerson)
Source: G3 (Bethesda). 2013 Apr 1;3(4):709–17. doi: 10.1534/g3.112.005124 (PMC3618357; doi:10.1534/g3.112.005124)
Supplement: Supporting Information [file supp_g3.112.005124_FigureS6.pdf]

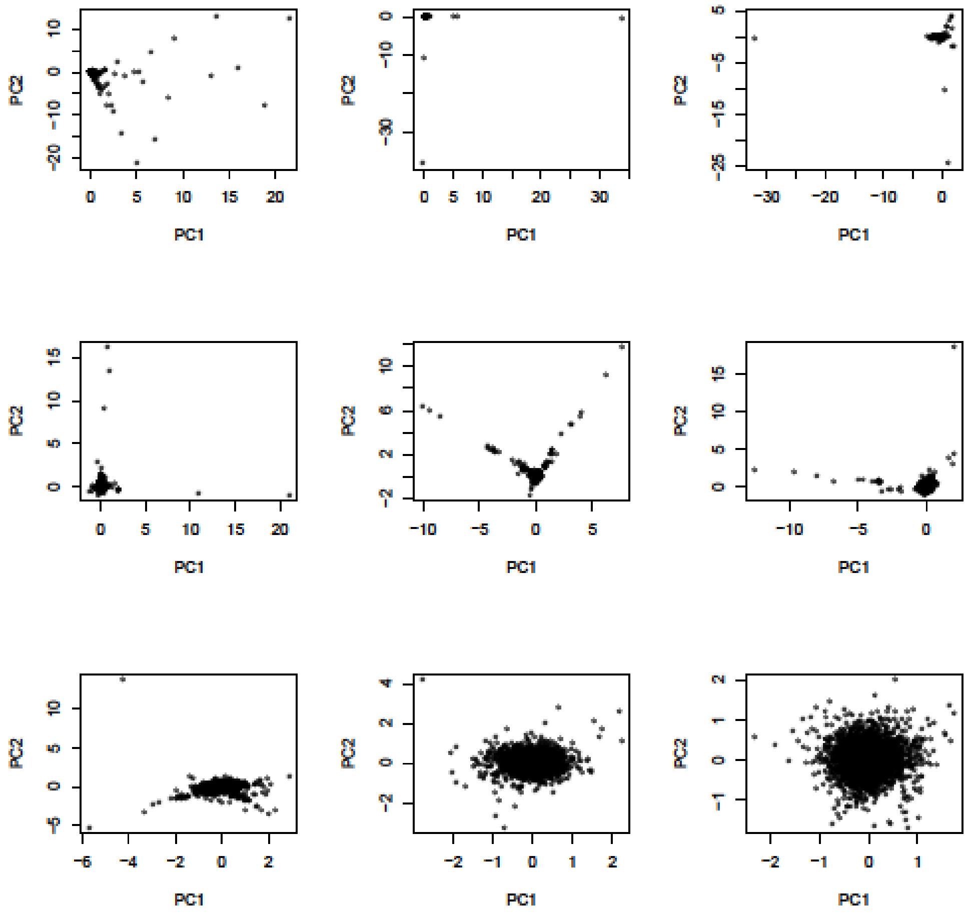

Figure S6 Correspondence analysis plots after nine iterations of removing outliers in the empirical mackerel data that satisfied the threshold $\sqrt{(PC1 + PC2)} > 2$ where *PC1* and *PC2* are the first and second principal components. Iterative steps are from top left to right moving down rows. The last plot 8 shows a cluster ball of genotypes after removing 116 genotypes from 5413 genotypes. One more iteration (not shown) removed 4 additional points.
