# Supplementary material for: Linkage Disequilibrium Estimation of Effective Population Size with Immigrants from Divergent Populations: A Case Study on Spanish Mackerel (Scomberomorus commerson)
Source: G3 (Bethesda). 2013 Apr 1;3(4):709–17. doi: 10.1534/g3.112.005124 (PMC3618357; doi:10.1534/g3.112.005124)
Supplement: Supporting Information [file supp_g3.112.005124_FigureS7.pdf]

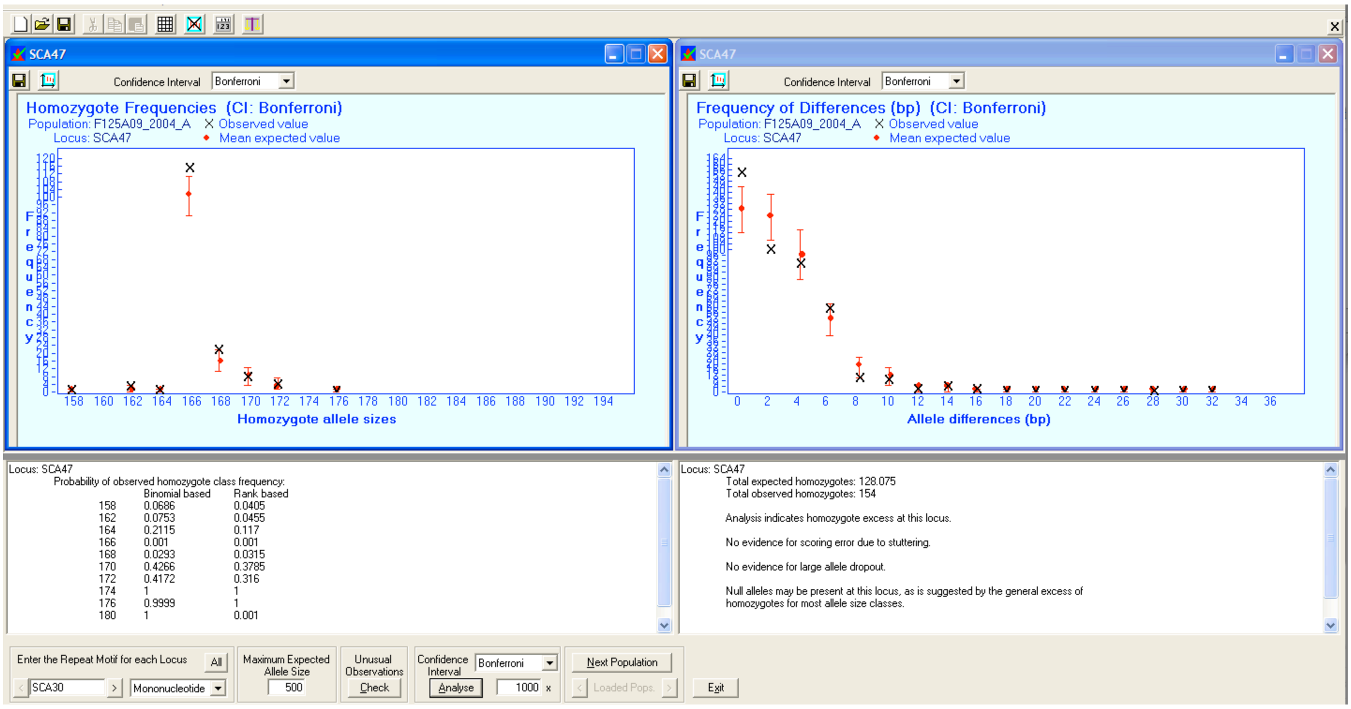

Figure S7 Graphical output from Microchecker software showing observed (X) and expected (red vertical bars) frequency of homozygotes (left panel) and heterozygotes (right panel) for 500 genotypes from 2004 collected adjacent to Darwin.
